# PHAROS—PHysical Assistant RObot System

**DOI:** 10.3390/s18082633

**Published:** 2018-08-11

**Authors:** Angelo Costa, Ester Martinez-Martin, Miguel Cazorla, Vicente Julian

**Affiliations:** 1 ALGORITMI Center, University of Minho, 4704-553 Braga, Portugal; 2 RoViT, University of Alicante, 03690 San Vicente del Raspeig (Alicante), Spain; ester@ua.es (E.M.-M.); miguel.cazorla@ua.es (M.C.); 3 Departamento Sistemas Informáticos y Computación, Universitat Politècnica de València, 46022 Valencia, Spain; vinglada@dsic.upv.es

**Keywords:** robot assistant, deep learning, cognitive assistant, elderly physical exercise, human exercise recognition, gesture recognition, ambient assisted living

## Abstract

The great demographic change leading to an ageing society demands technological solutions to satisfy the increasing varied elderly needs. This paper presents PHAROS, an interactive robot system that recommends and monitors physical exercises designed for the elderly. The aim of PHAROS is to be a friendly elderly companion that periodically suggests personalised physical activities, promoting healthy living and active ageing. Here, it is presented the PHAROS architecture, components and experimental results. The architecture has three main strands: a *Pepper robot*, that interacts with the users and records their exercises performance; the *Human Exercise Recognition*, that uses the Pepper recorded information to classify the exercise performed using Deep Leaning methods; and the *Recommender*, a smart-decision maker that schedules periodically personalised physical exercises in the users’ agenda. The experimental results show a high accuracy in terms of detecting and classifying the physical exercises (97.35%) done by 7 persons. Furthermore, we have implemented a novel procedure of rating exercises on the recommendation algorithm. It closely follows the users’ health status (poor performance may reveal health problems) and adapts the suggestions to it. The history may be used to access the physical condition of the user, revealing underlying problems that may be impossible to see otherwise.

## 1. Introduction

According to United Nations [1], the society is experiencing a great demographic change known as *ageing society*. Indeed, 13% of the world’s population is now elderly (i.e., people 60 years of age and older), while this percentage is predicted to increase to approximately 25% in 2050. This ageing phenomenon may impact on several socio-economic issues like the family sustainability or the economic growth [2]. In addition, ageing is a detriment to the person’s health (e.g., sensory impairment, diminished mobility) and, consequently, their autonomy. This dependency may force older adults to move to a care facility, what could result in depression, social isolation and/or even greater dependency in completion tasks [3]. For that reason, there is a growing demand for technology supporting the elderly at home.

In this respect, due to the wide variety of elderly needs, different solutions have been proposed in the literature. The most basic assistance can be provided with a system tele-operated by a professional. That is, for instance, the case of *InTouch Health* [4]. In this project, a mobile medical platform (i.e., RP-7 robot) remotely operated by a doctor is proposed. This robot is able to take the patient’s pulse, scan vital signs, take pictures and even read case notes. All this data is sent to the *tele*-doctor with the aim to advice medical staff on potentially life-saving actions if necessary. On the contrary, when health monitoring is required, health smart homes may be a solution. These residences equipped with sensors, actuators and/or biomedical monitors, collect data around the clock such as environmental conditions, habitant’s vital signs and/or their health condition (e.g., [5,6,7,8,9,10]). Going a step further, Ambient Assisted Living (AAL) paradigm processes that collected data in order to provide personalised assistance and a tailored experience. This can be achieved through automated or self-initiated medication reminders, management tools [11,12] or lost key locators [13], among others.

Focusing on cognitive decline, physical activity may be beneficial in preserving cognition at late life [14,15]. Actually, aerobic activity and strength exercises are strongly recommended to keep autonomy with ageing. Nevertheless, doing exercise implies adapting to a fixed session schedule and, sometimes, moving to a care facility to attend the corresponding sessions, what could inconvenience the elderly. As a solution, Bleser et al. [16] proposed *PAMAP* (Physical Activity Monitoring for Ageing People), a home-based platform for physical activity supervision and motivation. So, in the case of elder population, the system uses the television as an interface to set the exercises to be done (defined by a healthcare professional) as well as to provide feedback on the overall quality of the person’s performance. For this last issue, a group of sensors capturing person’s motions must be strategically worn during doing the exercises. In addition, taking into account the movement variability in exercise performance due to person’s physical limitations, a training phase is required. In particular, user’s motions for each exercise are recorded while a therapist is monitoring its performance. In this way, a personalised reference is created for performance evaluation.

Alternatively, a robotic assistant could be used. For instance, Matsusaka et al. [17] developed *TAIZO*, a small humanoid robot that can execute a number of callisthenics routines. So, from a voice command or keypad inputs, *TAIZO* performs the asked exercises while repeating pre-recorded motion explanations. In this case, the system is just an actuator since no observing action of the elderly’s performance is made. Going a step further, Gadde et al. [18] used *RoboPhilo* as an interactive personal trainer. Basically, the robot is in charge of showing and monitoring the user’s physician-prescribed exercise program. For that, the robot waits for a user’s presence. Then, it asks for user’s consent (i.e., waving one hand overhead) and, after that, the exercise routine starts. That is, *RoboPhilo* starts mimicking the first scheduled exercise while vocally explains the different body movements. The next step consists of monitoring user’s performance, analysing their timing and form. When the user’s motions are correct, the humanoid robot congratulates the user and continues with the next exercise. Otherwise, *RoboPhilo* repeats the exercise movements and instructions until the user correctly performs it. At the end of the interaction cycle, a verbal feedback about the overall user’s performance is provided. This system has several limitations. For instance, the user should be positioned at the centre of the video frame at the optimal distance (determined by user’s height). In addition, a good performance is only obtained under good light conditions (enough light is required for accurate hand/face detection). On their behalf, Görer et al. [19] presented an autonomous exercise tutor for elderly people. With that aim, the first stage involves a learning session with the therapist. During this session, the robot learns the person’s movements by means of imitation. At the same time, the therapist can provide the system with a verbal description accompanying each movement and/or the whole exercise. In the second stage, the NAO robot [20] behaves as an exercise tutor by repeating the learnt movements, whereas tracking and analysing user’s motions. As previously, the robot starts the session with a greeting and a consent request. After that, each exercise from the stored program is verbally introduced. Then, the robot reproduces the learnt movements. Next, the robot observes the user’s motions with the aim of evaluating their imitation. This process is repeated until the exercise program is completed. So, before concluding the session, the robot gives an overall performance score. The main drawbacks of this system include the loss of user’s progress since each user is identified by name, which can be repeated among the system’s users. In addition, the provided feedback omits the user’s physical capabilities because a fixed feedback is associated with a certain score range. Moreover, the exercise program was designed for an average, healthy elderly person, making it inappropriate for the majority of elder community. Finally, the user’s performance cannot be studied when the person is seated because the skeleton data are unreliable.

Despite the wide variety of state-of-the-art approaches, no appropriate system is available for assisting elderly in doing physical exercise at home. So, this paper introduces *PHAROS*, a physical assistant robot system aiding seniors in their daily physical activities and promoting their practice at home. PHAROS uses innovative methods of recommendation and exercises’ monitoring. The recommendation takes into account the elderly’s likes and health condition without requiring the caregivers guidance. The exercises’ monitoring uses novel Deep Leaning methods (like RNN, C2R and GRU) to identify accurately the body movements and position, using a robot camera. So, with these features, PHAROS is able to intelligently guide an elderly person to perform a physical exercise, allowing that elderly person to be more independent and pro-active.

Thus, this paper is organised as follows: Section 2 introduces PHAROS’s architecture, presenting its several components that are described in detail in Section 3 and Section 4; experimental results are presented and discussed in Section 5, while Section 6 concludes this work.

## 2. PHAROS Architecture

**Figure 1:**
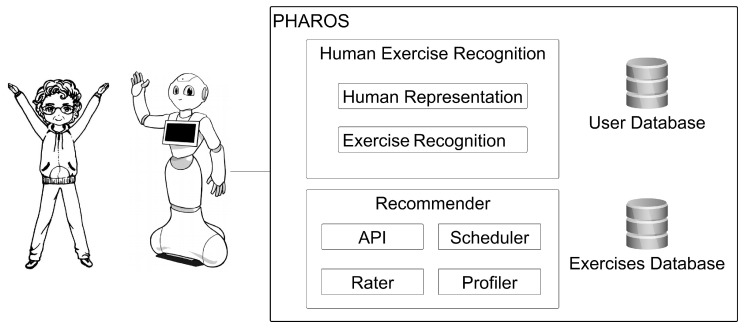
The PHAROS architecture.

PHAROS is an interactive robot platform designed for assisting the elderly in their daily physical activities at home. PHAROS architecture is divided into two different modules (see Figure 1). The *Recommender* recommends at a scheduled time activities that each user enjoys and is able to perform; and the *Human Exercise Recogniser* uses advanced Artificial Intelligent methods to identify human poses in real-time, verifying if they are correct. These two modules are specially designed to be implemented in a robot platform. Thus, the proposed robot platform should be friendly, intuitive and proactively assistive. In addition, it should be accepted by the elderly, what is crucial due to their systematic sceptical attitudes towards robots in their care. These requirements lead us to use a Pepper robot [21], a kindly, human-shaped robot with high levels of acceptance of the elderly [22], although any other robot could be used. With the use of the Pepper as the visual and physical interface with users, it initially interacts with the elder for his/her identification through its camera. Once the user is identified, PHAROS determines the most suitable physical exercises for him/her based on his/her physical limitations. From this information, it schedules a daily tailored physical series so that it is continuously being adapted by the *Rater* module to the elder’s evolution and his/her health status. So, the *Recommender* and *Human Exercise Recogniser* feed each other with information of the environment and user actions and are in constant communication. PHAROS differentiates itself from others robot platforms through carefully monitoring the users performing an exercise and evaluating if it was correctly performed or not, suggesting exercises for a healthy living. Moreover, with the history of performances, the caregivers are able to visualise if there is a decline of the ability to do certain exercises, which can reveal progressive physical and/or cognitive problems.

On its behalf, the *Scheduler* module triggers the corresponding reminder at the fixed time. For that, Pepper verbally warns the user about his/her daily healthy activities. Once the user’s attention is captured, PHAROS verbally describes the exercise to be done, while it is visually showed in Pepper’s screen. At this point, Pepper’s camera provides the visual input to the *Human Exercise Recogniser*. This module is responsible for monitoring and assessing the user’s performance of each exercise done. Finally, this data is sent to the *Recommender* with the aim of updating the information about the user’s health status and, accordingly, his/her daily scheduled physical activities.

## 3. Recommender (Rc)

The *Recommender*’s goal is to suggest activities to the user that will improve his/her physical and mental health. At a fixed time, it recommends the user a tailored physical exercise, helping him/her to keep active and in shape. Physical exercises are regarded as great tools to keep elderly people active as well as to improve their health condition and prevent future illnesses [14,15].

The *Rc* has a database of exercises that are beneficial to the users, classified by intensity and restrictions. For each user it is created a set of exercises that he/she is able to perform. Thus, the recommendation is personalised taking into account the physical and cognitive capacity of the user. The *Rc* operates through web-services that help to keep PHAROS free of proprietary systems. It uses REST services [23], exposing an API that receives queries and responds to them accordingly.

One of the most important features is the rating system. To avoid repetition, and thus boredom, an evolutive rating system was implemented to frequently update the rating of the exercises when parameters change. The aim of this system is two fold, on the one hand, it promotes the exercises the users perform better, while on the other hand it warns the caregivers about problems in performing certain exercises.

In the following subsections, *Rc* components illustrated in Figure 1 are explained.

### 3.1. User Registration

Integrated in the API, *user registration* is responsible for the identification of the user. The client application (Pepper Robot, Android application, browser application, etc.) registers the user in the system by sending an REST GET message. This is similar to a login system, where the system spawns a new service specific to that user. This service is also responsible for scheduling a new exercise at a personalised rate. The rate may be fine tuned to each user, e.g., every 4, 8, 12 or 24 h.

### 3.2. Exercise Recommendation

After the user is registered, the *Rc* is able to reply queries about that specific user. In this case, the *Rc* replies to queries about if there is any exercise to recommend. It uses a rating process (described in Section 3.4) to suggest personalised exercises. This may result in one of two responses: information about the exercise that was recommended, or a standardized *not available* response when no recommendation is available at that time.

### 3.3. Exercise Information Reception

Also integrated in the API, the *Rc* is able to receive information about the exercise performed by the user. The PHAROS *Human Exercise* recogniser (explained in Section 4) monitors the activity performed by the user, estimating the percentage of completeness of the exercise, i.e., how much and how well the user has done that exercise. That information is provided to the *Rc* that updates its internal records. The rating process is explained in more detail in Section 3.4.

### 3.4. Exercises Rating

Due to the health conditionings the users have, it is likely that they have fluctuations in the exercises’ performance. Furthermore, this can be aggravated by common problems like broken bones or muscular problems, which often occur to elderly people due to their fragility. Aiming at monitoring the evolution of the users (increasing and decreasing their health condition), it is necessary an algorithm that changes the suggestions to promote specific exercises over others. To solve this problem, the most effective way is to give the exercises a weight value that correlates to the user performance that is also available to evolve with the user.

To achieve this personalised recommendation, the *Rc* has a rating system that ranks the exercises taking into account the user performance, the suggestion frequency and the time periods between each suggestion.

The *Rc rating* algorithm was built upon the Glicko2 rating system, a detailed explanation of the Glicko2 algorithms can be found in [24]. Glicko2 is mostly known to be used in Chess championships and other type of games (most notably in computer games) due to its evolutive characteristics. The algorithm takes into account the win/loss ratio, the volatility of the players and time intervals. Explained briefly, Glicko2 is able to sustain high volatility (when players have a high or low score periods after perceived stability) without changing drastically the player’s rating, as well as normalising the player’s rating after a prolonged period of time without playing (the player’s rating slowly decreases over time, unlike the usual drastic drops of average-based algorithms). Thus, the algorithm sets a low-fluctuation, low-decreasing players’ rating, which is optimal for the *Rc* rating system. To this, the *Rc* considers exercises as players and each suggestion as a match.

#### 3.4.1. Rating Algorithm

Although the *Rc rating* is based on the concept of Glicko2 (explained in [24]), its algorithm has been extensively changed to be properly adapted to the new context. Like other rating systems, the Glicko2 tends to steadily increase (or maintain) the classification of the winner players, as they usually keep wining, which is unusable for the *Rc*. To keep the users interested, the *Rc* should suggest variate exercises, but at the same time, have a preference for exercises that are better performed by the users. People prefer novel tasks (to avoid boredom) but not too much or little challenge for them. That is, the *Rc* should have a set of exercises that closely follows this pattern.

**Figure 2:**
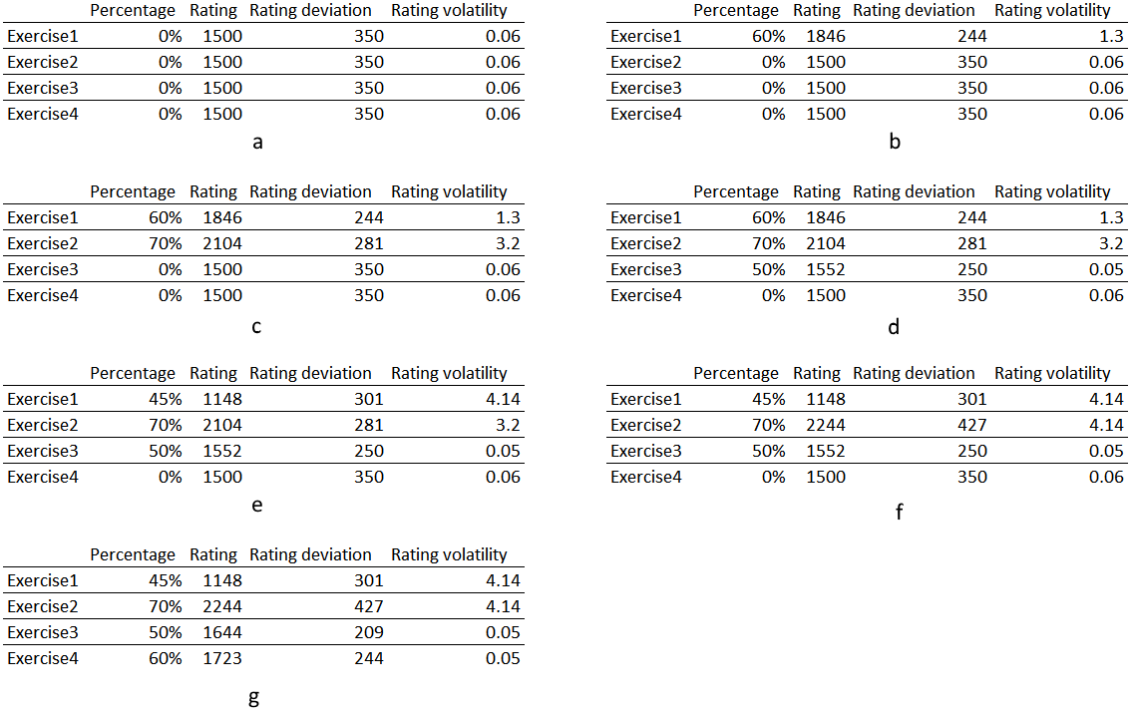
The outcome of the rating process and the self-correcting exercise suggestion. The fields are: the percentage of completeness, the exercise rating (1500 is the base value), the rating deviation (the confidence interval), and the rating volatility (the degree of expected fluctuation of the rating).

The suggestion process follows this flow:

1. For each user, the exercises he/she can perform are selected. This filter is supervised by formal caretakers that specify what exercises each user is able to perform without being a risk for his/her health. The filter is set initially when a user is introduced in the PHAROS system, and it is configurable at any time (see Figure 2a).
2. Each exercise possesses a rating or is given one (as shown in Figure 2a). Due to being personalised for each user, each exercise has a specific rating for each user, e.g., *Exercise*1 can have a rating of 1865 points for *User*1 and 1470 for *User*2.
  - In case of any exercise is unrated (first *Rc* execution or a new exercise introduced by the caregiver in the user profile), it is given the base rating of 1500 points and a small deviation and volatility values (Figure 2a). This means that most of the time, the *Rc* will not recommend this exercise to the user unless the rest of the exercises have lower classification, which is highly improbable.
  - The two previously recommended exercises are removed from the recommendation pool (Figure 2b and rest). This counteracts the bias towards high performed exercises. As stated before, winning exercises (the ones that are chosen over others) tend to continue to be chosen as winners (apart from changing health conditions of the users) due to their stable increasing rating. Thus, to introduce variance, the algorithm removes the previously performed exercises, avoiding exercise repetition and the creation of a small group of activities that is highly rated over the others.
  - The exercise with the highest rating is then selected and saved locally, available for the clients via the API. When the clients recall that information, it is replied with the complete information about the exercise, guaranteeing maximum compatibility with the clients.
3. After performing the exercises, the *Rc* waits for the performance values (percentage of completeness). When these values are received, the *Rc* rates the exercises (explained in Section 3.4.1.1). This process is done in the following way (illustrated in Figure 2):
  - The history about the proposed exercises is retrieved.
  - The “loser” exercises (the ones that were not selected to be performed) are separated in three groups in relation to the percentage received. The exercises that have better performance are classified as *over*, the ones that have similar values are classified as *same* (with a fluctuation of over and under 2 percentage points), and the ones that have lower percentage values are classified as *under*.
  - These events are then rated in relation to the received percentage (i.e., the exercise performed). Using a game-related language (Glicko2) the *over* exercises are evaluated as winners, the *same* exercises are evaluated as ties, and the *under* are evaluated as losers.
  - The resulting value is saved as the new exercise rating of that specific user. This rating is used in the next exercise suggestion.
  - The outcome of this process is a variation of the exercises rating. This asserts an evolutionary process that constantly evolves according to the user’s ability and health status.
4. The *Rc* enters in a sleep state until the scheduler restarts it.

##### 3.4.1.1. Rating Procedure

Figure 2 represents a trace of a probable rating procedure of the exercises of *User1*. Figure 2a shows the initial conditions in the exercises for *User1*. It can be observed that the values are the same for all exercises. This is due to the exercises not being initialised previously, being the initial state for all users. Randomly, *Exercise1* was suggested and attained a 60% of completeness, showed on Figure 2b. As this percentage was above all the others, the rating is higher, and, as a consequence of having only one iteration, the volatility also increases (meaning that a high fluctuation of the rating is expected in future iterations). Next, the *Rc* suggests the *Exercise2* (as the algorithm stops the two previous suggestions of being suggested again). In this execution, *User1* has a performance of 70% (Figure 2c). Thus, *Exercise2* is well over the others and the rating of *2104* is a proof of that. In the next suggestion, the *Exercise3* is chosen, but in this iteration the user only had a 50% completeness (Figure 2d). Therefore, the rating reflects that result, being lower than the exercises 1 and 2. In the following iteration, *Exercise1* is selected, but the *User1* has performed it poorly, as it is showed in Figure 2e. Thus the rating falls sharply, even lower than *Exercise4*, getting a high value of volatility (due to its inconsistency). Next, the *Exercise2* is suggested as it is the highest rated one (Figure 2f). The user gets the same percentage of completeness, thus the rating reflects this consistency, increasing only slightly. Finally, Figure 2g shows the results of two iterations, first the suggestion of *Exercise3* that yet again had 50%. In the second iteration only *Exercise4* could be chosen (exercises *2* and *3* are out of the exercise pool). The user performed well (60%) being placed as second in terms of rating.

As it can be observed in Figure 2f, after a few executions the rating values tends to follow the ability of the users to perform them, thus auto-correcting according to their overall performance. This has a positive side effect, it reveals the progression of the health condition of the user. A threshold can be set that warns the caregivers of possible health-related problems, when fast decaying values are detected. This the task of the *Profiler* (Figure 1), to find patterns that affect the user’s health. Furthermore, the caregivers can access the user’s history, being able to access how the exercises are impacting the users, and change the list of exercises the user is able to perform.

The *Rc* relies on the information that is sent by the Human Exercise Recognition. Without it, the *Rc* is unable to know if the users have done the suggested physical exercises, and how well they have being performed.

## 4. Human Exercise Recognition

Human action recognition has become extremely popular in recent years as a result of the vertiginous growth in social applications. In this context, the classic computer vision approaches based on RGB data could make the system miserably fail when facing illumination changes or intra-class variations, that are quite common in real scenarios. So, with the aim of overcoming visual failure issues, a human representation encoding and characterising human’s attributes from visual perception data is required as an early step.

Despite the wide literature in this research topic, the existing approaches can be broadly divided into two groups: representations based on local features (i.e., keypoints in the spatio-temporal space) [25,26]; and, skeleton-based representations, where a small number of representative joints encode the whole body configuration (e.g., [27,28]). However, the high computational cost and the inability of representing multiple individuals in the same scene make the methods based on local features unsuitable for the task at hand. As a consequence, a human representation based on 3D skeleton information with a high frame rate is required.

**Figure 3:**
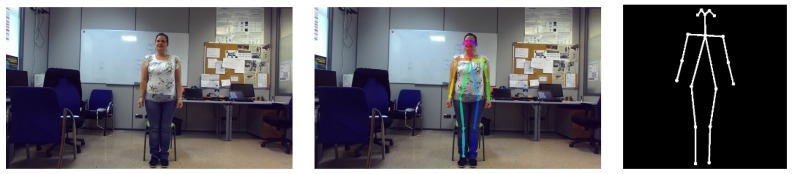
A sample of the 3D human skeleton extraction by using *Openpose* [29,30]. The left image corresponds to the captured frame; the middle one shows the estimated skeleton on the original image; and, the last one represents the resulting 3D human skeleton.

In particular, *Openpose* [29,30] is used in this work. Basically, it is a bottom-up approach for real-time multi-person pose estimation. So, this two-branch multi-stage Convolutional Neural Network (CNN) outputs a 18-keypoint body skeleton for all the people in the image. For that, human parts are predicted by means of confidence maps and combined through joint associations such that an articulated system of rigid segments connected by joints is generated for each person. As shown in Figure 3, the estimated joints are used to build a new 224 × 224 image where only human skeletons are kept. In this way, visual processing is focused on human actions, avoiding errors because of confusing background elements.

Given that doing a physical exercise may be described as a sequence of body motions, its recognition can be formulated as a sequence classification. Thus, from a series of visual input over time, the system must be able to robustly identify the exercise performed and send this information to the recommender system.

In this context, Deep Learning techniques drew a great attention. Specifically, Recurrent Neural Networks (RNNs) are mainly used to model the temporal dependencies between the skeletal data. Nevertheless, the dependency relations among the skeletal joints in the spatial domain are not considered despite their discriminative role for action classification. In this sense, Convolutional Neural Network (CNN)-based approaches are able to extract that spatial information form the skeleton data.

**Figure 4:**
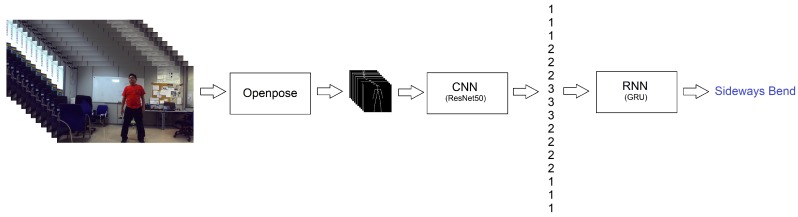
C2R architecture, which combines a CNN based on ResNet50 [31] with a RNN composed of GRU, to properly recognise the physical exercise done.

Aiming at properly exploiting spatio-temporal information, we proposed a combined neural network (C2R): a CNN based on ResNet50 [31] is used to firstly classify the human motion observed in each input image and, then, a chunk of estimated pose classes feeds into a RNN composed of a Gated Recurrent Unit (GRU) [32] to adaptively recognise each performed physical exercise (see Figure 4). So, the CNN part assigns a label per frame, while the RNN part returns a single label for an exercise video sequence.

GRU-based neural networks have been successfully applied to temporal data. In addition, GRUs are more efficient and less memory requiring than its RNN counterparts. On its behalf, ResNets improve the performance on the training set, which is a prerequisite to do well on the validation and test sets. This results in an easy way for a residual block to learn the identity function by adding skipped connections. Therefore, the combination of both architectures joins their advantages leading to a structure able to improve the movement recognition and, as a consequence, the exercise recognition in a more efficient way. In addition, this combination allows the system to exploit spatio-temporal information, which is crucial to achieve the required accuracy.

**Figure 5:**
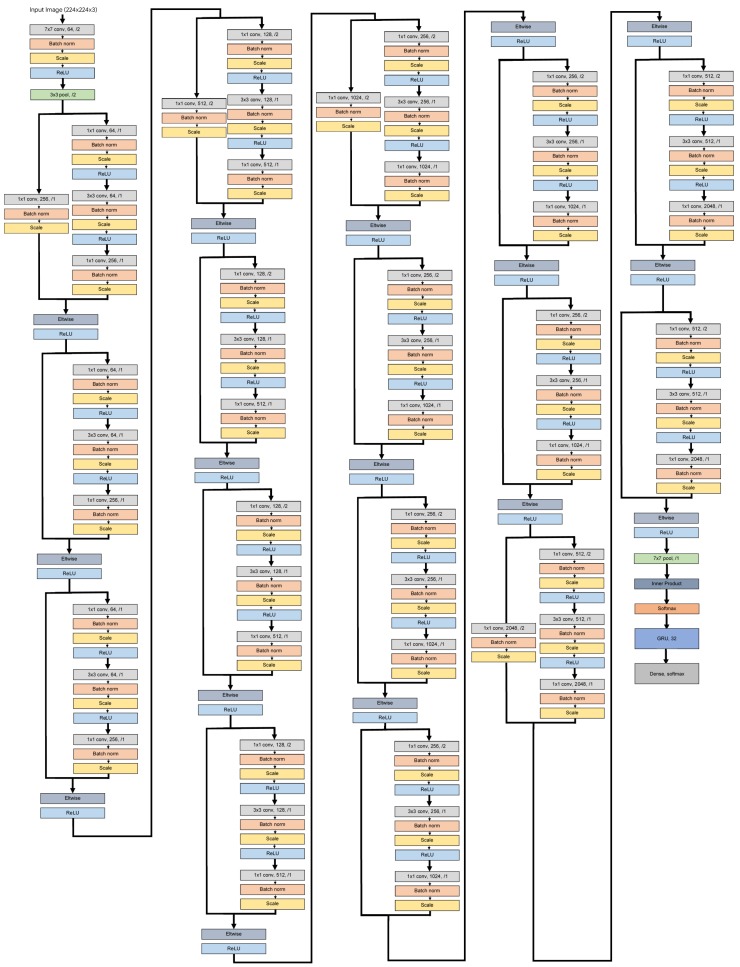
Layer architecture of C2R combining a ResNet50, a layer with an GRU and a dense layer with *softmax* activation.

Therefore, C2R is composed of 52 layers: 50 layers corresponding to the ResNet50, a layer with an GRU with 32 units and a dense layer with *softmax* activation (see Figure 5). In our experiments, the logarithmic function was used as loss function (or objective function) given that our final goal is knowing the performed action and its completeness (encoded as categories). In addition, the efficient gradient descendent algorithm adam [33] was used for optimization because it is a broader adoption for deep learning applications in computer vision, while classification accuracy is set as the metric since it is a classification problem. Note that all the required parameters were set to their default values.

**Figure 6:**
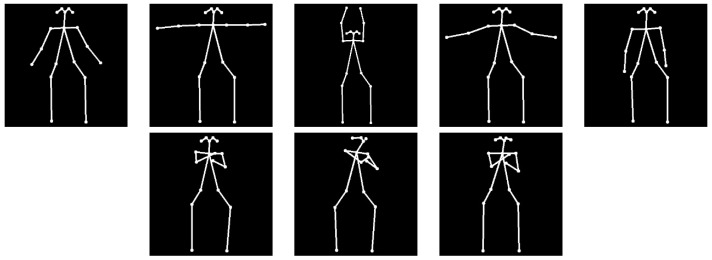
Human skeleton sequence encoding the required human positions to successfully do the *arm raises* exercise (**top**) and the *upper body twist* exercise (**bottom**).

A key issue is the length of the video sequence corresponding to any physical exercise. In fact, this variability mainly depends on two factors. On the one hand, different physical exercises involve a different number of human poses. So, for instance, the *arm raises* exercise needs five human poses, whereas the upper *body twist* exercise only requires three (see Figure 6). On the other hand, the restricted elder’s mobility considerably influences in the exercise duration. As a solution, the input sequences are truncated and padded so that all the input data has the same length for a correct modelling (zero values are learnt as no information elements). In this way, exercise sequences are represented by same length vectors, required to perform the computation, although they are different in terms of content. In particular, the experimental captured video sequences vary in length from 3 frames to 47 frames. So, the video sequence length for the RNN was set to the maximum length, that is, 47.

## 5. Experimental Results

The exercise program held in our experiments corresponds to the one suggested by the British National Health Security (NHS) for older people [34]. This program is divided into four exercise groups: sitting, flexibility, strength and balance. So, an elder’s exercise program includes a number of exercises from each group according to their age and their physical limits.

With the aim of validating PHAROS, the *Recommender* logic and rating procedure is assessed with few *personas* [35,36]. For that, a small elderly community was emulated. Due to space constraints, a user (John) is selected as a representative elder person. John is a healthy user, although he has a slight leg problem (i.e., difficulty in lifting his legs and walking medium distances). Taking this information into account, the *Rc* generates a first exercise recommendation. This recommendation is adapted each day according to John’s capabilities and the received completeness values since these values measure the John’s difficulty in performing each suggested physical exercise. Note that the number of daily physical exercises can vary due to several reasons like elder’s health status.

Assuming that one exercise is done per day, Table 1 shows the John’s recommendations during 100 days. Table 1 is divided into three columns (reduced from the complete information for simplicity): the *Iteration* column denotes the number of the iteration in natural order; the *Exercise* column shows the name of the exercise recommended (and performed) just as in NHS [34]; and, the last column, labelled as *% completed*, indicates the completeness value got from the *human exercise recogniser*, that is, how well John does the whole exercise.

Table 1 is a great example of the *Rc* operation. So, two features of *Rc* discussed in Section 3 are present in this table. In particular, they are the auto-correcting exercises rating and the detection of health issues from the percentage history. In John’s case, they can be observed in the following way:

- **Exercises distribution:**Table 1 shows the distribution of the exercises in a natural order. The first iterations (from 1 to 30) show a high diversity of exercises, while from thereon some repetition in exercises is observable. The reason behind this is that in the first iterations the rating of the exercises variation is low, making any exercise eligible. After this initial period, the difference between ratings starts to be substantial, promoting the exercises that receive higher percentages.
- **Health issues identified:** Despite John’s leg issues, some leg exercises have been recommended. An interesting pattern occurs with the *Step Up* exercise, the percentages are: 77.2%, 68.3%, 70.1%, 47.1%, 78.6%, 26.1%, 10%. This reveals that there is a clear decrease in the ability to perform this exercise, what may reveal a health problem. Another pattern is showed by the *Sideways Bend*, which has continuously low percentage values. This can be the outcome of one of two problems: physical problems affecting the exercise performance or poorly performing the exercise. It is clear that this is a problem unable to overcome by PHAROS, thus the assistance of a caregiver is required.

As above-mentioned, the completeness exercise evaluation is provided by *Human Exercise Recognition*. With the aim of evaluating the C2R’s performance, several subjects (seven in total) were recorded doing the 21 proposed physical exercises in a lab space. So, all the video sequences were manually labelled with the physical exercise performed (*chest stretch*, *upper body twist*, *hip marching*, *ankle stretch*, *arm raises*, *neck rotation*, *neck stretch*, *sideways bend*, *calf stretch*, *sit to stand*, *mini squats*, *calf raises*, *sideways leg lift*, *leg extension*, *wall press up*, *bicep curls*, *sideways walking*, *simple grapevine*, *heel to toe walk*, *one leg stand*). At the same time, each video sequence was sampled video at 15 frames per second, obtaining individual frames to be labelled with their corresponding human pose. Note that each human pose is assigned to a unique pose class and, consequently, different exercises sharing human poses (e.g., *arm raises* and *neck stretch*) provide a way to assess the robustness of the proposed CR2 approach for human pose recognition and human action recognition. In this dataset, the duration of an action ranges from 3 frames to on the order of 50 frames.

Then, the C2R was trained. For that, the first step was taken an image dataset by means of the Pepper’s colour visual system. This visual system is composed of two generic, identical video cameras located in the forehead that provide RGB, 2560 × 1080 images at 5 frames per second. Then, these images were processed by *Openpose* and, the resulting images, were manually labelled. After that, the CNN based on ResNet50 [31] was trained taking as input 224 × 224 × 3 the resulting skeleton images. As above-mentioned, these images correspond to a cropped, skeleton-centred *Openpose* output. In this way, the human pose is clearly represented at each frame, what helps in classifying it. This is specially important since a pose class involves more than one human pose. The reason lies in the fact that elderly’s physical condition affects exercise performance. As a solution, the pose classes (mainly bordering ones) include a wide range of limb positions, what minimises the difference between the last and the first human poses of two consecutive pose classes. For example, in the case of *raise arms* exercise, the “middle” arm position includes from 60 to 99 degrees, while the “top” arm position covers from 100 to 180 degrees.

So, with this pose classification, the C2R convolutional module was trained on 1915 skeleton images and validated on 1641 samples representing 9 pose classes (*sit upright with crossing arms reaching shoulders*; *sit upright with crossing arms reaching shoulders and upper body turned left*; *sit upright with crossing arms reaching shoulders and upper body turned right*; *sit upright held on the sides of the chair*; *sit upright held on the sides of the chair with left leg lifted*; *sit upright held on the sides of the chair with right leg lifted*; *sit upright with arms by sides*; *sit upright with arms up to middle torso height*; *sit upright with arms over head*). Note that the subjects used for training were different from those used for validation.

The training during 100 epochs leads to an accuracy of 99.9% for training and 87.7% over test data. As illustrated in Table 2 and Table 3, the most confused pose classes are those corresponding to the *sit-upright-with-crossing-arms-reaching-shoulders* poses, where the subtle torso turn is the key feature to be properly distinguished. On the contrary, the missclassification of classes *sit-upright-with-arms-up-to-middle-torso-height* and *sit-upright-with-arms-over-head* is due to the small difference in the arms position between them.

Next, the whole video sequences are used to train the C2R recurrent part. More precisely, each exercise sequence has been decomposed into its corresponding vector of pose classes, which is the required input for the GRU-based neural network. However, the current status of our dataset lacks enough samples to achieve a good performance. As a consequence, data augmentation has been applied.

A dataset analysis revealed that each pose class takes place in 10 frames as maximum. From this starting point, several exercise sequences have been generated by means of a fuzzy-logic process. That is, each pose class belonging to a specific physical exercise has been ordered included *n* times in the generated pose sequence, where *n* is a random integer between 1 and 10 set for each pose class and for each pose sequence. Thus, the C2R GRU-based network was trained over 159990 samples, while it was validated on 79995 exercise sequences. In this case, 15 physical exercises were considered: *upper body twist left*, *upper body twist right*, *hip marching left*, *hip marching right*, *arm raises*, *neck stretch left*, *neck stretch right*, *sit to stand*, *mini squats*, *sideways leg lift*, *leg extension*, *bicep curls*, *sideways bend*, *simple grapevine* and *heel to toe walk*.

The accuracy after 10 epochs is of 97.32% for training and 97.35% for test. Table 4 and Table 5 again highlights the difficulty in distinguishing between the two exercises related to *upper body twist* and both corresponding to *hip marching*.

Note that, with the aim of determining the *completeness* of each activity, different subsequences of an activity were also evaluated. That is, an activity is a sequence of human poses and, consequently, the *completeness* of an activity is given by the performed human poses. Thus, for instance, in the case of the *arm aises exercise*, the exercise is 100% complete if three poses are achieved in the right order: down arms–extended arms–rised arms–extended arms–down arms. On the contrary, when the person performs the sequence down arms–extended arms–down arms, the system outputs the right exercise (i.e., arm raises exercise), but 60% complete, which is the input for the Rc system.

**Figure 7:**
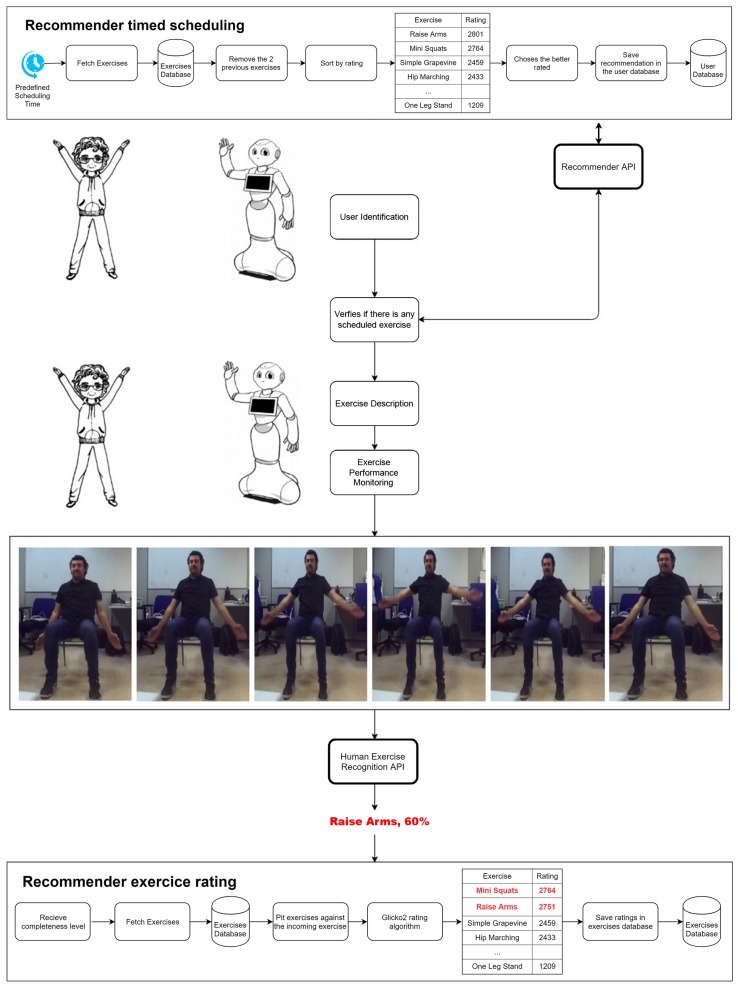
A real execution of PHAROS performance.

Finally, the whole PHARO’s performance was evaluated. As the real example illustrated in Figure 7, the starting point is the scheduled exercise program set by the Rc system. The *Recommender timed scheduling* process (presented in Section 3) is shown in the top part of the Figure 7. This process is composed of the following steps: (1) at a specific time (e.g., 10:00, 11:00, 15:00, etc.) the Rc triggers a new scheduling process; (2) upon starting, the user’s exercises are retrieved (they can be a subset of all the exercises); (3) the two previously performed exercises are removed from the pool of exercises, this is done to prevent exercises repetition bias; (4) then they are rated by their ranking (shown in the table inside the figure); (5) next, the highest rated is selected, in this case the *raise arms*, and saved in the user’s database, waiting to be recalled by the *Recommender API*.

The *Pepper* robot approaches to the user with the aim of reminding them the exercise time. When the user is properly identified by the robot through a visual face recognition, the robot platform queries the *Recommender API* for the first exercise to be performed (*raise arms* in this case). Thereafter, *Pepper* verbally explains the corresponding exercise while visually shows it on its screen. Meanwhile, the user’s monitoring is continuously taking images of the user’s performance. In that way, the user’s whole performance could be properly observed from its beginning whenever the user starts doing the corresponding physical exercise. As shown in the example (see image sequence in Figure 7), the user partially did the *raise arms* exercise (i.e., the arms-up pose is missing). This image sequence is the input to the *Human exercise Recognition API*. This module concludes that the performed exercise is *raise arms* with a completeness of 60%. This information is sent to the Rc system.

In the lowest part of Figure 7, the *Recommender exercise rating* can be found. Its task is to re-rate the exercises according to the received *completeness* value. It works in the following way: (1) through the *Recommender API* receives the *completeness* value of the suggested exercise, in this case, the *raise arms*; (2) the user’s exercises are retrieved from the database; (3) the exercises (*mini squats, simple grapevine, etc.*) are sorted using their most recent *completeness* value and separated into 3 groups and categorised as wins, ties, and losses; (4) then, with these groups, the *Glicko2* rating system is applied (the tendency of the increase/decrease rating is linked to the increase/decrease *completeness* value in relation to the previous one), this can be seen in red in the table where *raise arms* has now a lower rating than *mini squats*; (5) finally, the ratings are saved in the user’s database, ready to be used in the next iteration of the *Recommender timed scheduling*.

## 6. Conclusions

This paper has presented PHAROS, an interactive robot system designed for assisting elderly in their daily physical activities at home. For that, two different components are developed: the *Recommender* module and the *Human Exercise* recogniser. Broadly speaking, the elder is properly identified by using the *Recommender* API. After that, and according to the user’s physical limitations, the *Recommender* generates a tailored series of physical exercises and scheduled them in the user’s agenda. It is noteworthy that the proposed exercises are adaptively changed at least every day. This is necessary for two main reasons: avoiding elderly’s boredom and promote an appropriate elderly’s health fitting. So, in a timely manner, with the aim of promoting healthy living and active ageing, Pepper robot reminds the elderly their daily physical activity. Once the elderly attention is captured, PHAROS makes use of Pepper’s screen to show the next exercise to be performed and its audio system to verbally describe it. From then, Pepper’s camera provides the visual input to the *human exercise* recogniser. This module extracts the human skeleton by means of Openpose software and feeds it into the *exercise recogniser*, the C2R neural network. C2R combines a CNN based on ResNet50 to recognise human poses with a GRU-based RNN whose output is the corresponding exercise label together. This information is sent to the *Recommender* with the purpose of updating elderly’s health status and, consequently, their exercise recommendations.

PHAROS is a relatively recent development (being continuously improved) so that some of the features are still being tested and improved accordingly. Despite this, the current development shows promising results, which pushes forward the development of new features. PHAROS experiments show balanced suggestions validating two main goals: exercise variance (to keep users engaged) and personalization (to recommend physical exercises adjusted to the current elder’s health condition). Further tests with real users must be performed to evaluate the accuracy of the exercise selection algorithm. One predictable problem is the algorithm bias towards high ranking physical exercises. This issue can be solved with one of two approaches: manually by the caregivers, who receive a warning for manually scheduling a different exercise; or, automatically, the current implementation, where after few suggestions, a low-rated exercise is randomly selected. Additionally, the *Human Exercise* recogniser reaches 97.35% of accuracy in classifying physical exercises thanks to novel techniques as Deep Leaning to robustly extract information from the images captured by *Pepper*.

For future developments, we aim to improve the interaction of Pepper with the users. For instance, the lack of robot gestures mimicking the exercises, what can be misleading to the users. Moreover, further optimization of the *Human Exercise* recogniser could be achieved by means of building a larger dataset as well as using other mechanisms to make more distinguishable the unsuccessful physical exercises. In that way, PHAROS could gain accuracy in the classification and evaluation of each exercise. Another future development is the agenda synchronization between the users and Pepper. An assistant robot is still pricey (purchase cost, maintenance, etc.) and can be economically prohibitive to elderly with low income. The aim of this future development is that several users could use Pepper without interfering with the rest of the users, resorting to planning strategies.

## Figures and Tables

**Table 1:** Results of 100 iterations of the *Rc*.

Iteration	Exercise	% Completed
1	Step Up	77.22%
2	One Leg Stand	36.56%
3	Heel To Toe Walk	57.32%
4	Sideways Leg Lift	51.08%
5	Simple Grapevine	98.24%
6	Sideways Walking	46.45%
7	Bicep Curls	21.37%
8	Wall Press Up	38.53%
9	Leg Extension	69.89%
10	Calf Raises	26.92%
11	Mini Squats	70.21%
12	Sit To Stand	24.29%
13	Calf Stretch	50.37%
14	Sideways Bend	18.32%
15	Neck Stretch	73.81%
16	Neck Rotation	15.98%
17	Arm Raises	24.79%
18	Ankle Stretch	42.33%
19	Hip Marching	42.75%
20	Upper Body Twist	82.31%
21	Chest Stretch	92.06%
22	Neck Stretch	28.13%
23	Upper Body Twist	58.25%
24	Chest Stretch	29.05%
25	Mini Squats	34.71%
26	Leg Extension	34.74%
27	Hip Marching	94.54%
28	Calf Stretch	57.48%
29	Ankle Stretch	68.60%
30	Hip Marching	38.53%
31	Calf Stretch	4.36%
32	Ankle Stretch	61.89%
33	Simple Grapevine	31.51%
34	Heel To Toe Walk	65.25%
35	Ankle Stretch	62.11%
36	Sideways Leg Lift	40.53%
37	Heel To Toe Walk	97.96%
38	Ankle Stretch	33.68%
39	Sideways Leg Lift	49.15%
40	Heel To Toe Walk	21.79%
41	Wall Press Up	54.83%
42	Sideways Leg Lift	26.76%
43	Step Up	68.35%
44	Wall Press Up	37.55%
45	Sideways Walking	51.04%
46	Step Up	70.19%
47	One Leg Stand	57.18%
48	Sideways Walking	48.41%
49	Step Up	47.12%
50	One Leg Stand	47.33%
51	Sideways Walking	74.36%
52	Wall Press Up	88.32%
53	One Leg Stand	55.72%
54	Sideways Walking	75.57%
55	Step Up	78.66%
56	One Leg Stand	93.83%
57	Calf Raises	99.30%
58	Step Up	26.12%
59	One Leg Stand	75.13%
60	Calf Raises	96.80%
61	Bicep Curls	81.54%
62	One Leg Stand	68.40%
63	Calf Raises	21.20%
64	Bicep Curls	48.17%
65	One Leg Stand	88.30%
66	Arm Raises	88.16%
67	Bicep Curls	41.90%
68	Sit To Stand	49.46%
69	Arm Raises	29.93%
70	Bicep Curls	66.74%
71	Sit To Stand	63.15%
72	Sideways Bend	9.02%
73	Bicep Curls	11.09%
74	Sit To Stand	41.86%
75	Neck Rotation	59.92%
76	Simple Grapevine	80.48%
77	Sit To Stand	71.88%
78	Neck Rotation	94.45%
79	Simple Grapevine	77.62%
80	Ankle Stretch	63.68%
81	Neck Rotation	79.62%
82	Simple Grapevine	53.08%
83	Ankle Stretch	40.03%
84	Leg Extension	54.56%
85	Simple Grapevine	67.17%
86	Ankle Stretch	44.95%
87	Hip Marching	78.48%
88	Simple Grapevine	16.73%
89	Ankle Stretch	13.37%
90	Hip Marching	88.51%
91	Sideways Leg Lift	31.45%
92	Calf Stretch	63.53%
93	Hip Marching	38.82%
94	Wall Press Up	74.01%
95	Calf Stretch	70.55%
96	Mini Squats	30.17%
97	Wall Press Up	63.58%
98	Calf Stretch	77.22%
99	Step Up	10.01%
100	Wall Press Up	62.58%

**Table 2:** Confusion matrix corresponding to C2R-CNN training evaluation with 100 epochs when 9 pose classes are considered.

165	1	0	0	0	0	0	0	0
0	153	0	0	0	0	0	0	0
1	0	160	0	0	0	0	0	0
0	0	0	266	0	0	0	0	0
0	0	0	0	131	0	0	0	0
0	0	0	0	0	55	0	0	0
0	0	0	0	0	0	187	0	0
0	0	0	0	0	0	0	457	0
0	0	0	0	0	0	0	0	339

**Table 3:** Confusion matrix corresponding to C2R-CNN test evaluation with 100 epochs when 9 pose classes are considered.

109	0	5	0	0	0	0	0	0
25	3	11	0	0	0	0	31	0
45	0	50	0	0	0	0	0	0
0	0	0	129	7	3	1	0	0
0	0	0	1	40	10	0	0	0
0	1	0	8	2	28	1	0	0
0	1	0	0	0	0	212	23	0
0	0	0	0	0	0	11	526	6
0	0	0	0	0	0	0	10	342

**Table 4:** Confusion matrix corresponding to C2R training evaluation with 10 epochs when 15 physical exercises are considered.

10388	278	0	0	0	0	0	0	0	0	0	0	0	0	0
1913	8753	0	0	0	0	0	0	0	0	0	0	0	0	0
0	0	10492	174	0	0	0	0	0	0	0	0	0	0	0
0	0	1760	8906	0	0	0	0	0	0	0	0	0	0	0
0	0	0	1	10665	0	0	0	0	0	0	0	0	0	0
0	0	0	1	10	10649	6	0	0	0	0	0	0	0	0
0	0	0	1	2	21	10642	0	0	0	0	0	0	0	0
0	0	0	0	0	1	5	10660	0	0	0	0	0	0	0
0	0	0	0	0	0	0	0	10666	0	0	0	0	0	0
0	0	0	0	0	0	0	0	0	10666	0	0	0	0	0
0	0	0	0	0	0	0	0	1	0	10665	0	0	0	0
0	0	0	0	0	0	0	0	0	4	0	10637	25	0	0
0	0	0	0	1	0	0	0	0	1	0	28	10617	19	0
0	0	0	0	0	0	0	0	0	0	1	7	30	10628	0
0	0	0	0	0	0	0	0	0	0	0	0	0	0	10666

**Table 5:** Confusion matrix corresponding to C2R test evaluation with 10 epochs when 15 physical exercises are considered.

5193	140	0	0	0	0	0	0	0	0	0	0	0	0	0
905	4428	0	0	0	0	0	0	0	0	0	0	0	0	0
0	0	5245	88	0	0	0	0	0	0	0	0	0	0	0
0	0	891	4442	0	0	0	0	0	0	0	0	0	0	0
0	0	0	0	5333	0	0	0	0	0	0	0	0	0	0
0	0	0	0	10	5318	5	0	0	0	0	0	0	0	0
0	0	0	0	0	9	5324	0	0	0	0	0	0	0	0
0	0	0	0	0	0	0	5333	0	0	0	0	0	0	0
0	0	0	0	0	0	0	0	5333	0	0	0	0	0	0
0	0	0	0	0	0	0	0	0	5333	0	0	0	0	0
0	0	0	0	0	0	0	0	1	0	5332	0	0	0	0
0	0	0	0	1	0	0	0	0	1	0	5318	13	0	0
0	0	0	0	0	0	0	0	0	1	0	12	5310	10	0
0	0	0	0	0	0	0	0	0	1	0	2	26	5304	0
0	0	0	0	0	0	0	0	0	0	0	0	0	0	5333
